# Knowledge of complications of diabetes mellitus among patients visiting the diabetes clinic at Sampa Government Hospital, Ghana: a descriptive study

**DOI:** 10.1186/s12889-016-3311-7

**Published:** 2016-07-26

**Authors:** Yaa Obirikorang, Christian Obirikorang, Enoch Odame Anto, Emmanuel Acheampong, Emmanuella Nsenbah Batu, Agyemang Duah Stella, Omerige Constance, Peter Kojo Brenya

**Affiliations:** 1 Department of Nursing, Faculty of Health and Allied Sciences, Garden City University College (GCUC), Kenyasi Kumasi, Ghana; 2 Department of Molecular Medicine, School of Medical Science, Kwame Nkrumah University of Science and Technology (KNUST), Kumasi, Ghana; 3 Royal Ann College of Health, Department of Medical Laboratory Technology, Atwima Manhyia, Kumasi, Ghana

**Keywords:** Diabetic complication, Knowledge, Type 2 diabetics

## Abstract

**Background:**

Diabetes mellitus (DM) appears to be a global epidemic and an increasingly major non-communicable disease threatening both affluent and non-affluent society. The study aimed to determine the knowledge of diabetic complications among diabetes mellitus clients visiting the Diabetic Clinical at Sampa Government Hospital, Ghana.

**Method:**

This questionnaire-based descriptive study recruited a total 630 patients visiting the Diabetes Clinic at the Sampa Government Hospital. Structured questionnaire was used to obtain information such as socio-demographic and knowledge on complications of diabetes.

**Results:**

Out of a total of 630 participants, 325 (51.5 %) knew diabetic foot as the most common complication followed by hypertension 223(35.4 %), neuropathy 184 (29.2 %), hypoactive sexual arousal 160(25.4 %), arousal disorder 135(21.5 %), eye diseases 112(17.7 %), heart disease 58(9.2 %), and renal disease 34(5.4 %). Comprehensive assessment of level of knowledge on the complications showed that majority 378(60.0 %) of T2D patients did not have knowledge on diabetes complications, 169(26.9 %) had inadequate knowledge on diabetics complication while 82(13.1 %) had adequate knowledge. The risk factors associated with the level of knowledge of diabetic complications were female gender adjusted odd ratio (AOR) =2.31 (1.56–3.41) married participants AOR = 3.37 (1.44–7.93), widowed AOR = 2.98 (1.10–8.08), basic level of education AOR =0.18 (0.082–0.50), Junior High School (JHS) and above of education level AOR = 0.035(0.017–0.75), 5–9 years of T2D duration AOR = 0.31(0.018–0.57), ≥10 years T2D duration AOR = 0.042 (0.02–0.10) and urban dwellers AOR = 0.36 (0.22–0.68) respectively.

**Conclusion:**

Participants knew the individual complication of diabetic mellitus but lack an in-depth knowledge on the complications. Further expansion of diabetic educative programs like using mass media and involving national curriculum of education can improve self-regulatory awareness of diabetic complications which may reduce the morbidity and mortality of diabetic patients.

**Electronic supplementary material:**

The online version of this article (doi:10.1186/s12889-016-3311-7) contains supplementary material, which is available to authorized users.

## Background

Diabetes mellitus (DM) appears to be a global epidemic and increasingly a major non-communicable disease threatening both affluent and non-affluent society [1, 2]. More than 170 million people worldwide have diabetes, and this figure is projected to more than double by the year 2030, if the current trend is allowed to continue further [3]. The potential severity of increasing prevalence rate of diabetes on the African continent may be translated into severe economic burden, high morbidity and mortality rates that will surpass the ravages [4]. Diabetes prevalence studies in southern Ghana have recorded a steady increase. The earliest studies in the 1960s recorded 0.2 % prevalence in a population of Ghanaian men in Ho [5]. The crude prevalence of diabetes in general population was 6.3 % in the late 1990s in Accra, Ghana and the age adjusted prevalence of diabetes and impaired glucose tolerance (IGT) were 6.1 and 10.7 % respectively [6]. Individual diagnosed with diabetes tend to have an increased risk of stroke and heart diseases compared to the general population and increased incidence of retinopathy, peripheral nerve damage and renal problems [7].

Adequate knowledge of diabetes is a key component of diabetic care. Many studies have shown that increasing patient knowledge regarding disease and its complications have significant benefits with regard to patient compliance to treatment and to decreasing complications associated with disease [8]. Some research have been done into the knowledge and management including health education of the disease [9–13] but the prevalence of the disease still keeps rising in Ghana [14]. In spite of these researches and health education done on diabetes, most Ghanaians are still less knowledgeable about the complications associated with the disease according to the international Diabetes Federation report [15]. There is no published data with regards to the knowledge of diabetic complications in the Sampa district, Ghana. It was against this background that we determine the level of knowledge of diabetes mellitus complications among diabetics visiting the diabetic clinic at the Sampa Government Hospital, Ghana.

## Methods

### Study Design/Setting

An exploratory and descriptive study design was used to determine the knowledge on the complications of diabetes mellitus among diabetic patients visiting the diabetic clinic at Sampa Government Hospital from February to April 2015. Sampa is the district capital of Jaman North shares local boundaries with Tain District to the north-east, Jaman South District to the south and Berekum District to the south-east. The Jaman North has a total population of 83, 059 out of which Sampa account for a total population of 26,909 and has a total land area of 502 square km (Ghana statistical service, 2012). The Sampa Government Hospital is the only district hospital which serves the health needs of the residents and nationals from adjoining towns of neighboring La Cote D’Ivoire. The hospital currently has about 156 staffs comprising of one doctor who is the medical superintendent of the hospital, four medical assistants, one pharmacist, four midwives and 20 nurses. Playing a significant role in rendering a proximal service to clients, the facility has an Out Patient Department Laboratory services, Scan, Eye Clinic, ENT services, Public Health Services, Maternity Department, Theatre and a Diabetes Clinic which was started last year and managed by one medical assistant, two staff nurses and a nutritionist, accessed by diabetics on only Wednesdays. Annually, the hospital has an average attendance of 35,002 out of which 943 cases are diagnosed of diabetes.

### Study Population/Subject selection

Non-probability sampling technique was used to recruit six hundred and thirty (630) Type 2 Diabetic (T2DM) patients visiting the diabetic clinic. Structured questionnaires were used to obtain information from all study respondents. Structured questionnaire was based on the review of related journals [16–18]. The pre-test or pilot study was conducted among twenty (20) diabetics to ascertain the contents and clarity of the questionnaire. The questions were asked based on one-on-one interview with patients. Reliability coefficients ranging from 0.00 to 1.00, with higher coefficients indicating higher levels of reliability was used to determine the validity and the reliability of the questionnaire. The reliability coefficients for all the questions were 0.903. The entire questionnaire was available in English version but interviewed carefully with the proper translation of the official local language of the study population. The responses of the participants were translated back to English in the correct meaning as was interpreted. The structured questionnaire was divided into two sections with close-ended questions. Section A: involved questions that elicited information on socio-demographic variables of the patients such as age, gender, educational status, work pattern, family history of diabetes mellitus, duration of diabetes mellitus in the individual, treatment, dietary pattern, presence of any complications. Section B: included questions which assessed their knowledge on complications of diabetes mellitus and the kind of complications they know.

### Inclusion criteria

Type 2 diabetes patients who were not dumb or deaf, had no disabilities and had visited the hospital during the study period were included in the study.

### Exclusion criteria

Participants with type 1 diabetes, those who did not give consent as well as those with type 2 diabetes who were dumb or deaf were excluded.

### Knowledge of diabetes mellitus

Participant were said to have “adequate” knowledge of complication of Type 2 diabetes if they responded to at least three correct answers with a percentage score of 75–100 %; “inadequate “knowledge if they responded to at most one correct answer with a percentage score of <50 % and “no” knowledge (Don’t Know) if they did not know anything about the complications of diabetes (0 %).

### Data analysis

The data entry and analysis were performed using IBM statistical package for social science (SPSS) version 20. Descriptive statistics such as frequencies, percentage and charts were used. Chi-square or Fischer’s exact test statistical methods were used as appropriate to test association between categorical variables. Multivariable logistic regression analysis, adjusting for age and duration T2D was performed to predict factors associated with knowledge of diabetic complication. *P* value less than 0.05 was considered statistically significant difference.

## Results

### Socio-demographics characteristics of participants

Table 1 shows a total of 630 T2D patients were enrolled. The mean age of the general type 2 diabetic (T2D) patients in this study was 55.28 ± 14.71 years. A higher proportion (46.9 %) of them was between the ages of 40–59 years. Among the T2D subjects, there were more female (61.5 %) than male (38.5 %). Four hundred and sixty-five (465) representing 73.8 % were married. Out of a total of 630 subjects, 615 (97.7 %) had no socio-economic income, 495 (78.5 %) were unschooled, 383 (60.8 %) were Akans, 450 (71.5 %) had less than 5 years duration of T2D, while 344 (54.6 %) were from rural residency [Table 1].

**Table 1:** General Characteristic of Type 2 diabetic patients

Variables	Frequency	% Prevalence
Age (years) Mean ± SD	55.28 ± 14.71	
Age group		
<19	5	0.8 %
20–39	92	14.6 %
40–59	295	46.9 %
60–79	194	30.8 %
80–99	44	6.9 %
Gender		
Male	243	38.5 %
Female	387	61.5 %
Marital status		
Single	54	8.5 %
Married	465	73.8 %
Widowed	111	17.7 %
Economic income		
None	615	97.7 %
<500 (low)	5	0.8 %
500–1000 (medium)	5	0.8 %
>1000	5	0.8 %
Highest level of education		
None	495	78.5 %
Basic	43	6.9 %
JHS	58	9.2 %
SHS	24	3.8 %
Tertiary	10	1.5 %
Ethnicity		
Akan	383	60.8 %
Mole-Dagbani	247	39.2 %
Duration of diabetes (years)		
<5	450	71.5 %
5–10	126	20.0 %
11–15	39	6.2 %
16–20	15	2.3 %
Area of residence		
Rural	344	54.6 %
Urban	286	45.4 %

*SD* Standard deviation

### Knowledge of diabetic mellitus complications

Table 2 shows the proportion of participants’ response on the knowledge on diabetic complications. The most common diabetic complication known by diabetic patients was diabetic foot (51.5 %), followed by hypertension (35.4 %), neuropathy (29.2 %), hypoactive sexual arousal (25.4 %), arousal disorder (21.5 %), retinopathy (17.7 %), heart disease (9.2 %), and nephropathy (5.4 %) In general higher proportions of them were not knowledgeable on diabetic complications. Out of 630 patients surveyed, 606 (96.2 %) had no knowledge on sexual pain disorder, 528 (83.8 %) had no knowledge on heart disease, 450 (71.5 %) had no knowledge on nephropathy, neuropathy 427 (67.7 %), arousal disorder 422 (66.9 %), retinopathy 412 (65.4 %), hypoactive sexual arousal 412 (65.4 %) and diabetic foot 296 (46.9 %) [Table 2].

**Table 2:** Response of T2D participants on the complication of diabetes

Complications	Participants responses		
	Yes	No	Don’t Know
Hypertension	223 (35.4 %)	15 (2.3 %)	392 (62.3 %)
Heart Disease	58 (9.2 %)	44 (6.9 %)	528 (83.8 %)
Hypoactive sexual arousal	160 (25.4 %)	58 (9.2 %)	412 (65.4 %)
Arousal disorder	135 (21.5 %)	73 (11.5 %)	422 (66.9 %)
Sexual Pain disorder	0	24(3.8 %)	606 (96.2 %)
Retinopathy (Eye disease)	112(17.7 %)	106 (16.9 %)	412 (65.4 %)
Nephropathy	34 (5.4 %)	146 (23.1 %)	450 (71.5 %)
Diabetic Foot	325 (51.5 %)	9 (1.5 %)	296 (46.9 %)
Neuropathy	184(29.2 %)	19(3.1 %)	427 (67.7 %)

Values are presented as *N* (%): frequency (percentage)

### Level of knowledge of diabetic mellitus complications

**Fig. 1:**
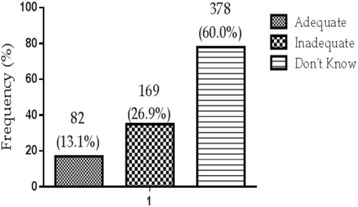
Overall knowledge score on complications of type 2 diabetes among diabetic patients

Figure 1 shows levels of knowledge of complication of Type 2 Diabetes In general majority 378 (60.0 %) of T2D patients did not have knowledge on diabetes complications. One hundred and sixty-nine (169) of them representing 26.9 % had inadequate knowledge on diabetics complication while a few of them 82(13.1 %) had adequate knowledge [Fig. 1].

### Association between levels of knowledge of diabetic mellitus complication and socio-demographic characteristics

Table 3 shows the association between age groups, gender, marital status, socioeconomic income, and duration of T2D, individual area of residency and level of understanding of diabetic complication. All patients 19 years and below knew nothing about diabetic complication. Higher proportion (22.2 %) of the elderly (80–99 year) had adequate knowledge on diabetic complications than 20–39 year (15.8 %), 40–59 year (13.1 %), and 60–79 (10.0 %). Age was not significantly associated with patient’s level of understanding of diabetic complications (*p* = 0.8198).

**Table 3:** Shows the association between age groups, gender, marital status, socioeconomic income, duration of T2D, individual area of residency and degree of understanding of diabetic complication

Variables		Level of understanding for diabetic complications			
	Total	Adequate (*n* = 82)	Inadequate (*n* = 170)	Don’t Know(*n* = 378)	*P*-value (*X*2, df)
Age group (years)					
<19	5	–	–	5 (100.0 %)	0.8198 (3.597, 8)
20–39	92	14(15.8 %)	34 (36.8 %)	44(47.4 %)	
40–59	295	39(13.1 %)	68(22.9 %)	188(63.9 %)	
60–79	194	19(10.0 %)	58(30.0 %)	116(60.0 %)	
80–99	44	10(22.2 %)	10(22.2 %)	24(55.6 %)	
Gender					
Male	243	44(18.0 %)	92(38.0 %)	107(44.0 %)	0.0131(8.675, 2)
Female	387	39(10.0 %)	77(20.0 %)	271(70.0 %)	
Marital status					
Single	54	10(18.2 %)	29(54.5 %)	15(27.3 %)	0.2125 (5.826, 4)
Married	465	58(12.5 %)	112(24.0 %)	295(63.5 %)	
Widowed	111	14(13.0 %)	29(26.1 %)	63(56.5 %)	
Socioeconomic income					
None	615	68(11.0 %)	169(27.6 %)	378(61.4 %)	0.0023 (20.41, 6)
<500 (low)	5	5(100.0 %)			
500–1000 (medium)	5	5(100.0 %)			
>1000 (high)	5	5(100.0 %)			
Highest level of education					
None	495	33(6.6 %)	114(23.1 %)	348(70.3 %)	<0.0001 (34.80, 8)
Basic	43	9(22.2 %)	14(33.3 %)	19(44.4)	
JHS	58	19(33.3 %)	24(41.7 %)	15(25.0 %)	
SHS	24	10(40.0 %)	14(60.0 %)		
Tertiary	10	10(100.0 %)			
Duration of T2D (years)					
<5	450	38 (8.6 %)	97(21.5 %)	315(69.9 %)	0.0076 (17.51, 6)
5–10	126	24(19.2 %)	39(30.8 %)	63(50.0 %)	
10–15	39	15(37.5 %)	15(37.5 %)	9(12.5 %)	
16–20	15	10(66.7 %)	5(33.3 %)		
Area of residence					
Rural	344	29(8.4 %)	92(26.8 %)	223(64.8 %)	0.206 (3.160, 2)
Urban	286	53(18.6 %)	78(27.1 %)	155(54.2 %)	

Values are presented as *N (%)* frequency (percentage), *(X2, df)* Chi-square, degree of freedom

More males (18.0 %) than female participants (10.0 %) had adequate knowledge on diabetic complications (*p* = 0.0131). Singles (18.2 %) followed by those widowed (13.0 %) and those who were married (12.5 %) had adequate knowledge on diabetic complications.

Marital status was not significantly associated with the degree of understanding of diabetic complications (*p* = 0.2125). Participants (100.0 %) who financially earned between 500 and 1000 Ghana cedis had adequate knowledge compared to 11.0 % of patients with no economic income (*P* =0.0023). All (100.0 %) participants who had completed tertiary education had adequate knowledge followed by those who had completed senior high school (40.0 %), junior high school (33.3 %), basic school (22.0 %) and those who were unschooled (6.8 %) (*p* <0.0001). The duration of patient’s diabetics was significantly (*p* = 0.0076) associated with the degree of understanding of diabetic complications. Approximately sixty seven percent (66.7 %) of participants with 16–20 years duration of diabetes had adequate knowledge on diabetic complications than to those with 11–15 year duration (37.5 %), 5–10 year (19.2 %) and below 5 years (8.6 %). There was no significant association between area of residence and the knowledge on diabetic complications (*p* = 0.2060). However, patients from the urban (18.6 %) had adequate knowledge than their counterparts from the rural settings (8.4 %) [Table 3].

### Factors associated with understanding of diabetic complications

Table 4 shows the multivariable logistic regression analysis of level of understanding for diabetic complications. Female gender adjusted odd ratio (AOR) =2.31(1.56–3.40), married participants AOR = 3.37 (1.44–7.93) and widowed participants AOR = 2.98(1.10–8.08) were significantly (*p* <0.05) associated with increased level of understanding of diabetic complications. Basic educational level AOR = 0.18 (0.082–0.50) JHS and above of educational level AOR = 0.04 (0.02–0.08), 5–9 years of T2D duration AOR = 0.31 (0.18–0.57), ≥10 years of T2D duration AOR = 0.42 (0.20–0.62) and urban dwellers AOR = 0.36 (0.22–0.68) respectively were significantly associated (*p* <0.05) with reduced level of understanding of diabetic complication [Table 4].

**Table 4:** Multivariable Logistic Regression analysis of level of understanding of diabetic complications

		Regression analysis Level of understanding of diabetic complications			
	Total	Adequate (*n* = 82)	Don’t Know(*n* = 378)	Adjusted OR (95 % CI)	*P*-Value
Age group (years)					
<40	97	14(15.8 %)	49(50.5 %)	1	
40–59	295	39(13.1 %)	188(63.9 %)	1.36(0.68–2.75)	0.361
Above 60	238	29(12.18 %)	140(58.82).	1.34 (0.67–2.82)	0.447
Gender					
Male	243	44(18.0 %)	107(44.0 %)	1.00	
Female	387	39(10.0 %)	271(70.0 %)	2.31 (1.56–3.41)	<0.0001
Marital status					
Single	54	10(18.2 %)	15(27.3 %)	1.00	
Married	465	58(12.5 %)	295(63.5 %)	3.37 (1.44–7.93)	0.0060
Widowed	111	14(13.0 %)	63(56.5 %)	2.98 (1.10–8.08)	0.0334
Socioeconomic income					
No income	615	68(11.0 %)	378(61.4 %)	1.00	
Yes income	15	15(100.0 %)	–	0.06 (0.003–0.98)	<0.0001
Highest level of education					
None	495	33(6.6 %)	348(70.3 %)	1.00	
Basic	43	9(22.2 %)	19(44.4 %)	0.18(0.082–0.50)	0.0009
JHS and above	92	39(42.39 %)	15(16.30.0 %)	0.04(0.02–0.08)	<0.0001
Duration of T2D (years)					
<5	450	38 (8.6 %)	315(69.9 %)	1.00	
5–9	126	24(19.2 %)	63(50.0 %)	0.31 (0.18–0.57)	0.0002
≥10	50	25(37.5 %)	9(12.5 %)	0.04 (0.23–0.62)	<0.0001
Area of residence					
Rural	344	29(8.4 %)	223(64.8 %)	1.00	
Urban	286	53(18.6 %)	155(54.2 %)	0.36 (0.22–0.68)	0.0001

Values are presented as *N (%)* frequency (percentage), *OR* odds ratio, *CI* confidence interval variables

## Discussion

This study aimed to determine the knowledge of complications of diabetes mellitus among diabetic patients visiting the diabetic clinic at the Sampa Government Hospital, Ghana.

In this study, the proportion of complication of type 2 diabetes commonly known by diabetic patients were diabetic foot (51.5 %), hypertension (35.4 %), neuropathy (29.2 %), hypoactive sexual arousal (25.4 %), arousal disorder (21.5 %), retinopathy (17.7 %), heart disease (9.2 %), and nephropathy (5.4 %).

A study conducted by Hoque and colleagues [17] among Indians visiting the Khulna Diabetic Centre, Bangladesh found heart disease (48.9 %) as the most common complication known by diabetic patient followed by cerebrovascular disease (15.2 %), renal disease (13 %), hypertension (5.4 %), and eye diseases (4.9 %) [17]. There was disparity of this study with another study where 53.5 % patients reported that heart disease was a potential complication of diabetes mellitus [19]. Another study observed that only 10 % of diabetic patients knew diabetic foot as a complication of diabetes [20]. Comparably, results of this present study differ from previous studies. The difference in response of patients’ knowledge on diabetic complication in this study compared to previous study may be explained by the difference in the diabetes education. Hoque and colleagues conducted their study among Indians population whiles our present study was among Ghanaian diabetic population. Reports indicate that differences in culture, race, and ethnic background may affect the pattern of knowledge on diabetic complication [21].

Increased response of patients’ knowledge on diabetic foot as the most common complication (Table 2) indicates that majority of participants had experienced this complication. In this study the decreased proportion of patients’ knowledge on heart disease compared previous study could be explained that either the diabetic patients had inadequate knowledge. Results from the present study showed that majority (60.0 %) of T2D patients did not have knowledge on diabetes complications. One hundred and sixty-five (165) of them representing 26.9 % had inadequate knowledge on diabetics complication while only 13.1 % of the sample population (*n* = 630) had adequate knowledge. Previous study conducted by Kavitha and Aruna, in 2014 among diabetic mellitus patients in India observed that 3.0 % of diabetics had adequate knowledge on acute complication of diabetic mellitus, which is consistent with the present study findings [22]. In another study where knowledge, attitude and practice were assessed among diabetic patients found that about 63.0 % did not know what diabetes and it complications were [23].

This study also observed a significant association between gender and the degree of knowledge for diabetic complications. Male diabetic participants had adequate knowledge of diabetic complications compared to their female counterparts. These results are consistent with the findings of Nisar et al. [24] conducted among diabetics living in Pakistan. Another study conducted in rural Northwest of Pakistan regarding knowledge of diabetes among patients showed that high proportion of males have better understanding of diabetes symptoms, signs and complication as compared to females [21]. These findings are also consistent with findings made by several other authors in a descriptive cross-sectional study [25, 26].

Another interesting finding of this study was the association between levels of education and the degree of patient’s knowledge on diabetic complications. It was observed that patients with tertiary education had significantly higher proportions in terms of adequate knowledge on diabetic complications. The results agrees with findings by Nisar et al., [24] who observed that diabetic patients with higher level of education were associated with greater knowledge of diabetic symptoms, risk factors, complications and presentation [24]. Another study showed that education had a significant role in diabetic awareness to keep correct blood glucose level [17]. These findings are consistent with several other studies among diabetic population [21, 26, 27]. There are no epidemiological studies in Ghana assessing the level of education and knowledge on diabetic complications. The significant association between education especially tertiary education and knowledge on diabetic complications is expected because, patients who had completed tertiary education might have attended workshop, conference, seminar and health talk on health-related issue.

This study also found a significant association between socio-economic income and the level of knowledge on diabetic complication. In this present study, diabetic patients irrespective of low, moderate or high economic income were significantly associated with adequate knowledge on diabetic complication compared to patients without economic income. A higher household income has been found to be associated with adequate knowledge on diabetic complications [28]. Despite the finding of this study Hoque and colleagues [17] observed no significant association between patients’ socioeconomic income and degree of understanding for diabetic complications.

Again, the significant association between knowledge on complication and duration of diabetes cannot be overlooked. This finding is expected because the longer the duration of diabetes the more knowledge patient has on diabetic complication. Longer duration of diabetes development are known factors and shown in numerous studies to be associated with the development and progression of chronic complications in diabetes [20].

A study in Pakistan indicated that type 2 diabetic patients who were urban dwellers were more knowledgeable than their counterparts residing in the rural area [29]. This finding is consistent with the present study finding where about 35.4 % of urban settlers compared to 8.4 % of rural settlers had adequate knowledge on diabetic complication. A similar finding was observed by Hoque, Islam, Khan, Aziz, & Ahasan, [17]. In this study, there was no significant difference in knowledge on diabetic complications between rural and urban dwellers probably because diabetes targeted education was lacking in both the groups. There is the need for urgent diabetic education among participants in these areas.

There are good evidences that foot complications are preventable by appropriate foot care and education programme [30]. In this study diabetic patients have successfully highlighted diabetic foot as the most common complication among diabetes in Sampa Metropolis.

The major limitation of this study was the sampling technique and the low literacy of the people. Their health literacy is likely low and perhaps they did not fully understand the questions and thus the findings of this study cannot conclusively represent the general diabetic patients in the Sampa government Hospital. However, some findings of this study concur well with other previous studies.

## Conclusion

In this study, the most common complication of type 2 diabetes known by diabetic patients was diabetic foot, followed by hypertension, neuropathy, hypoactive sexual arousal, arousal disorder, retinopathy, heart disease, and nephropathy. Higher proportion of the T2D patients did not have adequate knowledge on diabetic complications. Male gender, high income earners, higher level of education, and longer duration of T2D were significantly associated with degree of understanding for diabetic complications. It is incumbent on healthcare giver to provide early diabetic education regarding causes, management and preventive measures of diabetic complications. Organizing health education programmes as well as health outreaches on preventives measures such as adjusting to lifestyle and dietary modifications will enhance the level of knowledge of diabetic complications among diabetic patients.

### Abbreviations

DM, diabetes mellitus; IFG, impaired fasting glycaemia; IGT, impaired glucose tolerance; T2D, type 2 diabetes

## Additional file

Additional file 1: Data set on which conclusion of this manuscript was based upon. (XLSX 54 kb)
